# Automatic construction of rule-based ICD-9-CM coding systems

**DOI:** 10.1186/1471-2105-9-S3-S10

**Published:** 2008-04-11

**Authors:** Richárd Farkas, György Szarvas

**Affiliations:** 1 Research Group on Artificial Intelligence of the Hungarian Academy of Sciences, Aradi Vértanúk tere 1., Szeged, Hungary; 2 University of Szeged, Department of Informatics, Human Language Technology Group, Árpád tér 2., Szeged, Hungary

## Abstract

**Background:**

In this paper we focus on the problem of automatically constructing ICD-9-CM coding systems for radiology reports. ICD-9-CM codes are used for billing purposes by health institutes and are assigned to clinical records manually following clinical treatment. Since this labeling task requires expert knowledge in the field of medicine, the process itself is costly and is prone to errors as human annotators have to consider thousands of possible codes when assigning the right ICD-9-CM labels to a document. In this study we use the datasets made available for training and testing automated ICD-9-CM coding systems by the organisers of an International Challenge on Classifying Clinical Free Text Using Natural Language Processing in spring 2007. The challenge itself was dominated by entirely or partly rule-based systems that solve the coding task using a set of hand crafted expert rules. Since the feasibility of the construction of such systems for thousands of ICD codes is indeed questionable, we decided to examine the problem of automatically constructing similar rule sets that turned out to achieve a remarkable accuracy in the shared task challenge.

**Results:**

Our results are very promising in the sense that we managed to achieve comparable results with purely hand-crafted ICD-9-CM classifiers. Our best model got a 90.26% F measure on the training dataset and an 88.93% F measure on the challenge test dataset, using the micro-averaged *F*_β=1_ measure, the official evaluation metric of the International Challenge on Classifying Clinical Free Text Using Natural Language Processing. This result would have placed second in the challenge, with a hand-crafted system achieving slightly better results.

**Conclusions:**

Our results demonstrate that hand-crafted systems – which proved to be successful in ICD-9-CM coding – can be reproduced by replacing several laborious steps in their construction with machine learning models. These hybrid systems preserve the favourable aspects of rule-based classifiers like good performance, and their development can be achieved rapidly and requires less human effort. Hence the construction of such hybrid systems can be feasible for a set of labels one magnitude bigger, and with more labeled data.

## Background

The assignment of International Classification of Diseases, 9th Revision, Clinical Modification (ICD-9-CM) codes serves as a justification for carrying out a certain procedure. This means that the reimbursement process by insurance companies is based on the labels that are assigned to each report after the patient's clinical treatment. The approximate cost of ICD-9-CM coding clinical records and correcting related errors is estimated to be about $25 billion per year in the US [1]. There are official guidelines for coding radiology reports [2]. These guidelines define the codes for each disease and symptom and also place limitations on how and when certain codes can be applied. Such constraints include the following:

• an uncertain diagnosis should never be coded,

• symptoms should be omitted when a certain diagnosis that is connected with the symptom in question is present and

• past illnesses or treatments that have no direct relevance to the current examination should not be coded, or should be indicated by a different code.

Since the ICD-9-CM codes are mainly used for billing purposes, the task itself is commercially relevant: false negatives (i.e. missed codes that should have been coded) will cause a loss of revenue to the health institute, while false positives (overcoding) is penalised by a sum three times higher than that earned with the superfluous code, and also entails the risk of prosecution to the health institute for fraud.

The possibilities of automating the ICD-9-CM coding task have been studied extensively since the 1990s. Larkey and Croft [3] assigned labels to full discharge summaries having long textual parts. They trained three statistical classifiers and then combined their results to obtain a better classification. Lussier et al. [4] gave an overview of the problem in a feasibility study. Lima et al. [5] took advantage of the hierarchical structure of the ICD-9 code set, a property that is less useful when only a limited number of codes is used, as in our study.

Automating the assignment of ICD-9-CM codes for radiology records was the subject of a shared task challenge organized by the Computational Medicine Center (CMC) in Cincinatti, Ohio in the spring of 2007. The detailed description of the task, and the challenge itself, can be found in [6], and also online [7]. The most recent results are clearly related to the 2007 Challenge on Classifying Clinical Free Text, some of the systems that have been published so far can be found in [8], [9], [10] and [11]. Here 44 teams submitted well-formatted results to the challenge and, among the top performing systems, several exploited the benefits of expert rules that were constructed either by experts in medicine, or by computer scientists. This was probably due to the fact that reasonable well-formatted annotation guides are available online for ICD-9-CM coding and that expert systems can take advantage of such terms and synonyms that are present in an external resource (e.g. annotation guide or dictionary). Statistical systems on the other hand require labeled samples to incorporate medical terms into their learnt hypothesis and are thus prone to corpus eccentricities and usually discard infrequent transliterations or rarely used medical terms. While the CMC challenge involved a considerable but limited number of codes (there were 45 distinct labels used in the challenge dataset), the feasibility of constructing expert systems for hundreds or thousands of codes is not straightforward and undoubtedly time consuming if one wants to model all the possible inter-dependencies between labels. Thus in our study we examined how well top performing expert rule-based classifiers could be approximated via the extension of basic skeleton expert systems by machine learning methods. Such skeleton rule-based classifiers can be obtained automatically or semi-automatically (depending on how well the guide is structured in a textual format), directly from the publicly available ICD-9-CM coding guides. Table 1 shows how coding guides can be transformed into basic coding systems. Thus our goal in this article is to substitute the laborious process of manually collecting rare synonyms (which are not present in a coding guide), inter-label dependencies and common abbreviations from labeled data with training machine learning models that perform these steps. This approach exploits the advantages of expert systems, is able to handle rare labels effectively (using the information given in coding guides), and it is easier to apply even for a high number of labels as the more time-consuming steps of constructing rule-based ICD-9-CM coding systems are replaced by machine learning methods.

**Table 1:** Generating expert rules from an ICD-9-CM coding guide.

**CODING GUIDE**	**GENERATED EXPERT RULES**
**label 518.0**	**if document contains**
Pulmonary collapse	*pulmonary collapse***OR**
Atelectasis	*atelectasis***OR**
Collapse of lung	*collapse of lung***OR**
Middle lobe syndrome	*middle lobe syndrome*
Excludes:	**AND document NOT contains**
atelectasis:	
congenital (partial) (770.5)	*congenital atelectasis***AND**
primary (770.4)	*primary atelectasis***AND**
tuberculous, current disease (011.8)	*tuberculous atelectasis*
	**add label 518.0**

## Results

### Discovering inter-label dependencies

In order to discover relationships between a disease/illness and symptoms that arise from it, we applied statistical learning methods. For example, the presence of code *486* corresponding to *pneumonia* implied that the patient has certain symptoms like *786.2* and *780.6* (referring to *coughing* and *fever*). Since our initial rule-based system that simply implemented the instructions found in the ICD-9-CM guide lacked such information, it regularly overcoded documents with symptom labels. This kind of overcoding appeared in the form of false positive symptom labels in the output of the rule-based system.

This overcoding can be overcome by adding decision rules to the expert system to delete some symptom labels when specific labels corresponding to diseases are found. These extra decision rules can be produced manually. We found four rules good enough to worth adding to a rule-based system (with manual inspection of the data). These were:

• delete code *786.2(coughing)* when code *486(pneumonia)* is present,

• delete code *780.6(fever)* when code *486(pneumonia)* is present,

• delete code *786.2(coughing)* when code *493.90(asthma)* is present and

• delete code *780.6(fever)* when code *599.0(urinary tract infection)* is present.

Deriving such rules based on observations of the data itself is actually quite time-consuming, so we decided to test whether or not such rules could be induced automatically. To do this, we used the labels assigned by the initial rule-based system as features and trained a C4.5 decision tree classifier for each symptom label, treating the symptom false positive labels as the positive class and all other cases as negative examples. This way the decision tree learned to distinguish between false positive symptom labels and true positive ones. This statistical approach found five meaningful decision rules in the dataset, among which were all four rules that we enumerated above. The new rule was:

• delete code *788.30(incontinence)* when code *593.70(vesicoureteral reflux)* is present.

This fifth rule did not bring any improvement on the challenge test set (these two codes were never added to the same document). Because the four useful rules and the additional one that brought only a marginal improvement on the training dataset were found via our statistical approach – without inducing any detrimental disease-symptom relationships – we can say that this step of creating ICD-9-CM coding systems can be successfully automated.

The modeling of inter-label dependencies brought about a 1.5% improvement in the performance of our rule-based system, raising the micro-averaged *F*_β=1_ score from 84.07% to 85.57% on the training dataset and from 83.21% to 84.85% on the challenge test set.

### Collecting synonyms from labeled data

Although the available ICD-9-CM guides contain many useful synonyms, and incorporating them has the advantage of adding such phrases to the classifier model that are indicators of the corresponding label with a very high confidence, the coverage of these guides is not perfect. There are expressions and abbreviations which are characteristic of the particular health institute where the document was created, and physicians regularly use a variety of abbreviations. As no coding guide is capable of listing every possible form of every concept, to discover these infrequent keywords the examination of labeled data is necessary. The extension of the synonym lists can be performed via a manual inspection of labeled examples, but this approach is most laborious and hardly feasible for hundreds or thousands of codes, or for a lot more data than in the challenge. Hence this task should be automated, if possible. The effect of enriching the vocabulary acquired from coding guides is very important, and this step reduced the classification error by 30% when we built a system manually (the basic system with label-dependency rules had a 84.85% *F*_β=1_ score, while a similar system with manually enriched sets of synonyms performed slightly over 89%).

Since missing transliterations and synonyms can be captured through the false negative predictions of the system, we decided to build statistical models to learn to predict the false negatives of our ICD-9-CM coder. A token level Vector Space representation of the documents (token uni- bi- and trigrams were used) was applied here as a feature set. This way we expected to have the most characteristic phrases for each label among the top ranked features for a classifier model which predicted the false negatives of that label.

#### Training a C4.5 decision tree for false negatives

We tested a simple approach of building statistical classifiers to predict the false negatives (missed cases) of the basic rule-based system. Of course such predictions can be made by discovering terms that were missing from the synonym lists of the rule-based classifier.

We used a C4.5 decision tree learning algorithm for this task. The decision tree builds models that are very similar in structure to the rule-based system (simple if-then rules corresponding to the appearance or absence of uni-, bi- or trigrams of tokens). Thus such learnt models can be directly incorporated into the rule-based system or the classifier can be used in a cascade architecture after the rule-based system has performed pre-labeling.

With this approach we managed to extend the rule-based model for 10 out of 45 labels. About 85% of the new rules were synonyms (e.g. *Beckwith-Wiedemann syndrome, hemihypertrophy* for *759.89 Laurence-Moon-Biedl syndrome*) and the remaining 15% were abbreviations (e.g. *uti* for *599.0 urinary tract infection*). The procedure improved the overall micro-averaged *F*_β=1_ scores from 85.57% (training dataset) and 84.85% (challenge test dataset) to 90.22% and 88.925%, respectively. The system yielded better recall (89.96%) than precision (87.92%) on the challenge test set.

#### Iterative enriching using Maximum Entropy classifier

We examined the dictionary enriching task using Maximum Entropy models in an iterative way. We used the *P*(*false negative*) probabilities for each token level uni-, bi- or trigram feature as an indicator of feature relevance. We ranked all words and phrases according to their relevance on false negative predictions and added the most reliable keywords and phrases to the dictionary of the rule-based classifier. This procedure was repeated until the most significant feature brought fewer than 2 additional true positive predictions. With this approach we managed to extend the rule-based model for 9 labels. The set of terms acquired by this iterative method is twice as large as that obtained by the decision tree. Even so, the difference between their accuracies on the challenge test dataset is unquestionably below the level of significance. This approach improved the overall micro-averaged *F*_β=1_ scores from 85.57% (training dataset) and 84.85% (challenge test dataset) to 90.26% and 88.934%, respectively. The system yielded better recall (90.04%) than precision (87.85%) on the challenge test set.

## Discussion and conclusions

### Discussion

The CMC Challenge on Classifying Clinical Free Text Using Natural Language Processing demonstrated that expert rule-based approaches are competitive to, or even outperform, purely statistical approaches to the ICD-9-CM coding of radiology reports. On the other hand, the construction of systems that use hand-crafted decision rules would become more laborious and hard to accomplish when the number of codes involved in the task is a magnitude bigger than that used in the CMC challenge. To overcome this problem, we examined the possible ways of replacing certain phases of the construction of rule-based systems by statistical methods, while keeping the advantages of expert systems.

Our results demonstrate that, after the conversion of ICD-9-CM coding guides (which were originally designed for humans and are not machine readable), the major steps of building a high performance rule-based classifier for coding radiology reports can be replaced by automated procedures that require no human interaction. We studied two aspects of the construction of a purely hand-crafted rule-based system, namely the modeling of inter-label dependencies, which is a special characteristic of ICD-9-CM coding and the enriching of the synonym list of the rule-based system with rare transliterations and abbreviations of symptoms or diseases. The results of our experiments are summarized in Table 2. A webpage where all the systems described can be accessed and tested online is available at [12].

**Table 2:** Overview of our results.

	train	test
45-class statistical	88.20	86.69
Simple rule-based	84.07	83.21
Rule-based with label-dependencies	85.57	84.85
Hybrid rule-based + C4.5	90.22	88.92
Hybrid rule-based + MaxEnt	90.26	88.93
CMC challenge best system	90.02	89.08

All values are micro-averaged *F*_β=1_.

The *45-class statistical* row stands for a C4.5 classifier trained for single labels. The *CMC challenge best system* gives the results of the best system that was submitted to the CMC challenge. All our models use the same algorithm to detect negation and speculative assertions, and were trained using the whole training set (simple rule-based model needs no training) and evaluated on the training and the challenge test sets. The difference in performance between the 45-class statistical model and our best hybrid system (that is, using rule-based + MaxEnt models) proved to be statistically significant on both the training and test datasets, using McNemar's test with a *p* < 0.05 confidence level. On the other hand, the difference between our best hybrid model (constructed automatically) and our manually constructed ICD-9-CM coder (the CMC challenge best system) was not statistically significant on either set.

To perform these tasks with machine learning models, we trained classifiers to predict the errors of a basic rule-based system which relies just on the knowledge found in the coding guide. We trained C4.5 decision trees to predict false positive labels using the output of the rule-based system as features to discover disease-symptom relations, using pre-labeled training data. Here we found the same dependencies, and got the same improvement in performance, as that of a system with hand-crafted rules for inter-label dependencies.

To enrich the list of synonyms used by the rule-based system with additional phrases and abbreviations, we trained C4.5 and maximum entropy classifiers to predict the false negatives of the rule-based system using the Vector Space representation of the texts. These statistical models can be used in a cascade model following the rule-based system, or the most reliable keywords found can be incorporated as decision rules into the expert system. However, the difference in performance between these two different machine learning methods was below the statistical level of significance.

The extracted synonyms and abbreviations correlated well with those phrases added manually to the hand-crafted system. A small percentage of the phrases were clearly noise, that causing the systems to overfit on the training dataset – these systems achieved better performance on the training set than the hand-crafted system and performed somewhat worse on the evaluation set, see Table 2. The manual filtering of phrases proposed by the learning models could be performed in a few minutes, and this way more robust (and more similar to the hand-crafted) hybrid systems could be built with minimal effort. In our experiments we performed the major steps of the construction of a hand-crafted expert system using statistical methods. Evidently, the performance of the hand-crafted system is an upper bound on the performance that can be attained this way. We found that similar results could be achieved via statistical models by improving basic rule-based classifiers like those we obtained by an entirely hand-crafted system. The main contribution of the study described here is that such automatic systems can be constructed at a lower cost, with less human labour.

### Agreement rates

The results reported here are close to the performance that human expert annotators would achieve for the same task. The gold standard of the CMC challenge dataset is the majority annotation of three human annotators. The inter-annotator agreement statistics are shown in Table 3. We should mention here that the human annotators had no access to knowledge about the majority labeling, while models trained on the challenge dataset can model majority labeling directly. This way, human annotator agreement with majority codes should be higher if they had the chance of examining the characteristics of majority labeling. On the other hand, the annotators influenced the target labels as these were created based on their single annotations. This fact explains why all annotators have a higher agreement rate with the majority annotation than with other human annotators. It would be interesting to see the agreement rate of a fourth human annotator and majority codes, given that the human annotator could now examine the characteristics of the majority codes but have no direct effect on their assignment. This statistic would provide a better insight into the theoretical upper bound for system performance (the human performance) on this task.

**Table 3:** Inter-annotator agreement rates on the challenge train / test sets, in micro-averaged *F*_β=1_.

	A1	A2	A3	GS	BasicRB	Hybrid
A1	–	73.97/75.79	65.61/67.28	83.67/84.62	75.11/75.56	78.02/79.19
A2	73.97/75.79	–	70.89/72.68	88.48/89.63	78.52/78.43	83.40/82.84
A3	65.61/67.28	70.89/72.68	–	82.01/82.64	75.48/74.29	80.11/78.97
GS	83.67/84.62	88.48/89.63	82.01/82.64	–	85.57/84.85	90.26/88.93
BasicRB	75.11/75.56	78.52/78.43	75.48/74.29	85.57/84.85	–	–
Hybrid	78.02/79.19	83.40/82.84	80.11/78.97	90.26/88.93	–	–

A1, A2 and A3 refers to Annotators 1, 2 and 3 respectively. GS stands for gold standard labeling, while BasicRB represents our basic rule-based system that models inter-label dependencies. Hybrid denotes our hybrid rule-based + MaxEnt statistical model.

The significantly lower agreement between single human annotators shows that different health institutes probably have their own individual style of ICD-9-CM labeling. We also listed the agreement rates of annotators and the gold standard labeling with our basic rule-based system with label dependencies. This system can be regarded as a hypothetical human annotator in the sense that it models the ICD-9-CM coding guide an annotator should follow, not the gold standard labeling of the data itself. The fact that human annotators agree slightly better with this system than each other also proves that they tend to follow specific standards that are not neccessarily confirmed by official annotation guidelines. It is also interesting to see that majority labling has a significantly higher agreement with this system than single annotators. This observation seems to justify that majority coding of independent annotators indeed estimates ICD-9-CM coding guidelines better than single expert annotators.

All the above findings hold when we restrict the agreement evaluation to the 45 labels that appear in the gold standard. Agreement between human annotators remains comparable to their agreement with the the coding guide (basic rule-based, BRB system). Each of the annotators have one preferred partner with whom their agreement is slightly better than with the BRB system, and show definitely lower agreement with the other human annotation. The gold standard labeling agrees better with BRB than any single annotation by almost 3%, which also emphasises that majority annotation is capable of correcting mistakes and is better than any single human annotation.

### Error analysis

The current systems have certain limitations when compared to the ICD-9-CM coding of expert annotators. Take, for example, the following record from the training set:

***Clinical history:****None given*.

***Impression:****Normal chest*.

The annotators – given that the record itself contains nothing of relevance for any ICD code – then conclude that this must be report of a *routine chest x-ray* (*V72.5*) as these reports originate from a radiology department. Such complex inferences are beyond the scope of automated systems. Still, the obvious advantage of automated coding is that it is less prone to coding errors in simpler (and more frequent) cases. Some improvement, however, could be achieved by using a more sophisticated method to identify the scope of negation and speculative keywords than we applied here. Take, for instance, the following record:

***Clinical history:****Cough and fever*.

***Impression:****Right middle and probable right lower lobe pneumonia*.

The use of syntactic structure to determine the scope of negation and speculative keywords would allow the coding of pneumonia here. Our current system considers the token *pneumonia* as speculative, but in the second sentence *right middle* corresponds to pneumonia as well and is in a non-speculative context.

### Conclusions

The analysis of classification errors revealed that our results are quite close to the upper limit of performance that can be attained using the CMC challenge dataset. The similar results we obtained with two different classifiers and two different approaches used to extend the initial rule-based model also support this conclusion. The vast majority of classification errors are caused either by very rare cases (single specific usages not covered) or by not dealing with temporal aspects. The labeling of the dataset itself seems to be inconsistent regarding temporality, thus we think that there is little hope of building simple rule-based or statistical models that would detect past illnesses reported in the records and improve the overall system performance. We should add here that there were 23 records where our final system could not assign any code. As every medical record contains at least a symptom or a disease label, it would be worthwhile dealing with these cases.

Small improvements could also be achieved by using better models for negation and speculative cases or by incorporating richer lists of synonyms as the examples above make clear. Addressing these two tasks is what we plan to do in the future, but adding very rare terms would probably require the assistance of a physician to avoid overfitting on the labeled data.

## Methods

### Language processing for ICD-9-CM coding

In order to perform the classification task accurately, some pre-processing steps have to be performed to convert the text into a consistent form and remove certain parts. First, we lemmatized and converted the whole text to lowercase. For lemmatization issues we used the freely available Dragon Toolkit [13]. Next, the language phenomena that had a direct effect on ICD-9-CM coding were dealt with. As a final step, we removed all punctuation marks from the text.

According to the official coding guidelines, negated and speculative assertions (also referred as soft negations) have to be removed from the text as negative or uncertain diagnosis should not be coded in any case. We used the punctuations in the text to determine the scope of keywords. We identified the scope of negation and speculative keywords to be each subsequent token in the sentence. For a very few specific keywords (like *or*) we used a left scope also, that was each token between the left-nearest punctuation mark and the keyword itself. We deleted every token from the text that was found to be in the scope of a speculative or negation keyword prior to the ICD-9-CM coding process. Our simple algorithm is similar to NegEx [14] as we use a list of phrases and their context, but we look for punctuation marks to determine the scopes of keywords instead of applying a fixed window size.

In our experiments we found that a slight improvement on both the training and test sets could be achieved by classifying the speculative parts of the document in cases where the predicative texts were insufficient to assign any code. This observation suggests that human annotators tend to code uncertain diagnosis in those cases where they find no clear evidence of any code (they avoid leaving a document blank). Negative parts of the text were detrimental to accuracy in any case. Our final language processing method was the following:

1. Remove all tokens within the scope of a speculative or negation keyword. Classify the document.

2. If the document received no code in step 1, classify the document based on the speculative parts.

Here we made use of negation and speculative keywords collected manually from the training dataset. Speculative keywords which indicate an uncertain diagnosis were collected from the training corpus: *and/or*, *can*, *consistent*, *could*, *either*, *evaluate*, *favor*, *likely*, *may*, *might*, *most*, *or*, *possibility*, *possible*, *possibly*, *presume*, *probable*, *probably*, *question*, *questionable*, *rule*, *should*, *sometimes*, *suggest*, *suggestion*, *suggestive*, *suspect*, *unless*, *unsure*, *will*, *would*.

Negation keywords that falsify the presence of a disease/symptom were also collected from the training dataset: *cannot*, *no*, *not*, *vs*, *versus*, *without*.

The accurate handling of these two phenomena proved to be very important on the challenge dataset. Without the negation filter, the performance (of our best system) decreased by 10.66%, while without speculation filtering the performance dropped by 9.61%. We observed that there was a 18.56% drop when both phenomena were ignored. The above-mentioned language processing approach was used throughout our experiments to permit a fair comparison of different systems (all systems had the same advantages of proper preprocessing and the same disadvantages from preprocessing errors). As regards its performance on the training data, our method seemed to be acceptably accurate. On the other hand, the more accurate identification of the scope of keywords is a straightforward way of further improving our systems. Example input/output pairs of our negation and speculation handling algorithm:

1. **Input:***History of noonan's syndrome. The study is being performed to **evaluate** for evidence of renal cysts*.

**Output:***History of noonan's syndrome. The study is being performed to*.

2. **Input:***Mild left-sided pyelectasis, **without** cortical thinning or hydroureter. Normal right kidney*.

**Output:***Mild left-sided pyelectasis. Normal right kidney*.

Temporal aspects should also be handled as earlier diseases and symptoms (in case having no direct effect on the treatment) should either not be coded or distinguished by a separate code (like that in the case of code *599.0* which stands for *urinary tract infections*, and *V13.02* which stands for *history of urinary tract infections in the past*). Since we were unable to find any consistent use of temporality in the gold standard labeling, we decided to ignore the temporal resolution issue.

### Multi-label classification

An interesting and important characteristic of the ICD-9-CM labeling task is that multiple labels can be assigned to a single document. Actually, 45 distinct ICD-9-CM codes appeared in the CMC Challenge dataset and these labels formed 94 different, valid combinations (sets of labels).

There are two straightforward ways of learning multi-label classification rules, namely treating valid sets of labels as single classes and building a separate hypothesis for each combination, or learning the assignment of each single label via a separate classifier and adding each predicted label to the output set. Both approaches have their advantages, but they also have certain drawbacks. Take the first one; data sparseness can affect systems more severely (as fewer examples are available with the same set of labels assigned), while the second approach can easily predict prohibited combinations of single labels.

Preliminary experiments for these two approaches were carried out: machine learning methods were trained on the Vector Space representation (language phenomena were handled but the ICD-9-CM guide was not used). In the first experiment we used 94 code-combinations as the target class of the prediction and we trained 45 classifiers (for each code separately) in the second one. The procedures achieved micro-averaged *F*_β=1_ scores of 81.97% and 85.58%, respectively, on the training set, using 5-fold cross-validation. Based on these preliminary (and baseline) results we decided to treat the assignment of each label as a separate task and made the hypothesis that in an invalid combination of predicted labels any of them can be incorrect. Hence we did not attempt to post-process such outputs that did not occur in the training data.

### Building an expert system from online resources

There are several sources from where the codes of the International Classification of Diseases can be downloaded in a structured form, including [15], [16] and [17]. Using one of these a rule-based system which performs ICD-9-CM coding by matching strings found in the dictionary to identify instances belonging to a certain code can be generated with minimal supervision. Table 1 shows how expert rules are generated from an ICD-9-CM coding guide. The system of Goldstein et al. [11] applies a similar approach and incorporates knowledge from [17].

These rule-based systems contain simple if-then rules to add codes when any one of the synonyms listed in the ICD9-CM dictionary for the given code is found in the text, and removes a code when any one of the excluded cases listed in the guide is found. For example, code *591* is added if either *hydronephrosis, hydrocalycosis* or *hydroureteronephrosis* is found in the text and removed if *congenital hydronephrosis* or *hydroureter* is found. These expert systems – despite having some obvious deficiencies – can achieve a reasonable accuracy in labeling free text with the corresponding ICD-9-CM codes. These rule-based classifiers are data-independent in the sense that their construction does not require any labeled examples. The two most important points which have to be dealt with to get a high performance coding system are the lack of coverage of the source dictionary (missing synonyms or phrases that appear in real texts) and the lack of knowledge about inter-label dependencies needed to remove related symptoms when the code of a disease is added.

### The C4.5 Classifier

C4.5 is based on the well-known ID3 tree learning algorithm, which is able to learn pre-defined discrete classes from labeled examples. The classification is done by axisparallel hyperplanes, and hence learning is very fast. We chose to employ decision trees for two reasons. First, this algorithm is designed to handle discrete features (as in our situation) efficiently. Second, the learned models are human readable hence human experts can verify or modify them if they wish.

We used the freely available Weka package [18] and we constructed decision trees that had at least 2 instances per leaf, and used pruning with subtree raising and a confidence factor of 0.25 – which are default settings in the Weka package. The fine-tuning of the parameters could further improve performance.

### Maximum Entropy Classifier

Maximum Entropy Models [19] seek to maximise the conditional probability of classes, subject to feature constraints (observations). This is performed by weighting features to maximise the likelihood of data and, for each instance, decisions are made based on features present at that point, so maxent classification is quite suitable for our purposes. As feature weights are estimated in parallel, the maxent classifier is capable of taking feature dependence into account (for example, the bigrams *first uti* and *with uti* are downweighted as they are dependent on a strong and more general unigram, *uti*). Getting a good understanding of the role of the individual feature weights is not straightforward, hence we performed the following on each feature ranking subtask: an instance was created for each feature (just that particular feature occurred in the instance) and the predicted *P*(+) values for that instance were used as weights. In our experiments we made use of the OpenNLP maxent package [20], with smoothing and a 0.1 rate of smoothing observation.

## Competing interests

The authors declare that they have no competing interests.

## Authors' contributions

Author 1 and Author 2 conceived the method and prepared the manuscript. Author 2 implemented the rule-based initial system and its extensions, and the language processing method used. Author 1 implemented the statistical classifiers and the hybrid systems.
